# Comparison of seven anthropometric indexes to predict hypertension plus hyperuricemia among U.S. adults

**DOI:** 10.3389/fendo.2024.1301543

**Published:** 2024-03-08

**Authors:** Ye Li, Ling Zeng

**Affiliations:** ^1^Department of Critical Care Medicine, West China Hospital, Sichuan University, Chengdu, China; ^2^West China School of Nursing, Sichuan University, Chengdu, China

**Keywords:** anthropometric indexes, hypertension, hyperuricemia, NHANES (National Health and Nutrition Examination Survey), adults (MeSH)

## Abstract

**Purpose:**

This study aims to compare the association of hypertension plus hyperuricemia (HTN-HUA) with seven anthropometric indexes. These include the atherogenic index of plasma (AIP), lipid accumulation product (LAP), visceral adiposity index (VAI), triglyceride-glucose index (TyG), body roundness index (BRI), a body shape index (ABSI), and the cardiometabolic index (CMI).

**Methods:**

Data was procured from the National Health and Nutrition Examination Survey (NHANES), which recruited a representative population aged 18 years and above to calculate these seven indexes. Logistic regression analysis was employed to delineate their correlation and to compute the odds ratios (OR). Concurrently, receiver operating characteristic (ROC) curves were utilized to evaluate the predictive power of the seven indexes.

**Results:**

A total of 23,478 subjects were included in the study. Among these, 6,537 (27.84%) were patients with HUA alone, 2,015 (8.58%) had HTN alone, and 2,836 (12.08%) had HTN-HUA. The multivariate logistic regression analysis showed that the AIP, LAP, VAI, TyG, BRI, ABSI, and CMI were all significantly associated with concurrent HTN-HUA. The OR for the highest quartile of the seven indexes for HTN-HUA were as follows: AIP was 4.45 (95% CI 3.82-5.18), LAP was 9.52 (95% CI 7.82-11.59), VAI was 4.53 (95% CI 38.9-5.28), TyG was 4.91 (95% CI 4.15-5.80), BRI was 9.08 (95% CI 7.45-11.07), ABSI was 1.71 (95% CI 1.45 -2.02), and CMI was 6.57 (95% CI 5.56-7.76). Notably, LAP and BRI demonstrated significant discriminatory abilities for HTN-HUA, with area under the curve (AUC) values of 0.72 (95% CI 0.71 - 0.73) and 0.73 (95% CI 0.72 - 0.74) respectively.

**Conclusion:**

The AIP, LAP, VAI, TyG, BRI, ABSI, and CMI all show significant correlation with HTN-HUA. Notably, both LAP and BRI demonstrate the capability to differentiate cases of HTN-HUA. Among these, BRI is underscored for its effective, non-invasive nature in predicting HTN-HUA, making it a superior choice for early detection and management strategies.

## 1 Introduction

Hypertension (HTN) is a major risk factor for stroke, cardiovascular disease, and kidney failure, and is a leading cause of death globally (1, 2). It is estimated that by 2025, the prevalence of HTN will have increased by 60%, affecting 1.56 billion people (3). In the US, it is estimated that over 100 million people suffer from this common chronic condition (4, 5). Uric acid is the end product of purine metabolism in humans, and any disruption of purine metabolism can lead to increased uric acid levels and hyperuricemia (HUA). According to recent statistics, the incidence of HUA in the US stands at 21.2% among males and slightly higher at 21.6% among females (6). Research has demonstrated that 25-40% of people with high uric acid have untreated HTN (7). The meta-analysis showed a substantial association between serum uric acid levels and HTN, even when traditional risk factors were taken into account (8–10). HTN and HUA are major features of the metabolic syndrome, and they are important risk factors for cardiovascular disease. When HTN is combined with HUA, the damage to organs is usually more extreme than that caused by HTN alone (11–13).

Obesity is a medical condition in which the body accumulates too much fat, resulting in a disrupted metabolism and physiology (14). The figures from 2017-2018 show that the rate of this disorder in the US is increasing, as 42% of the population have a body mass index (BMI) of 30 or higher, and 9.2% have a BMI of 40 or more (15, 16). It is known that obesity can lead to HTN and HUA (17, 18). Adipose tissue inflammation and immune responses caused by obesity can lead to metabolic issues and insulin resistance, both locally and systemically (19). BMI is a widely accepted measure of obesity, yet it is not sufficient to determine the amount of visceral fat, dyslipidemia, and insulin resistance linked to obesity. Therefore, researchers have proposed new anthropometric tools that better reflect these characteristics, such as atherogenic index of plasma (AIP), lipid accumulation product (LAP), visceral adiposity index (VAI), triglyceride-glucose index (TyG), body roundness index (BRI), a body shape index (ABSI), and cardiometabolic index (CMI) (20–26).

While numerous studies have explored the correlation between various anthropometric indexes and either HTN or HUA (27–29), few have compared the predictive power of these indexes in patients with HTN-HUA. This research gap is particularly pronounced given the multitude of proposed anthropometric indexes. Moreover, to affirm the link between various anthropometric indexes and HTN-HUA, a large population sample is essential for validating extrapolated conclusions. Hence, this study aims to discern the predictive power of anthropometric indexes - AIP, LAP, VAI, TyG, BRI, ABSI, and CMI - in patients with HTN-HUA, with a view to identifying the most accurate predictors.

## 2 Materials and methods

### 2.1 Study population

This study utilizes data extracted from the National Health and Nutrition Examination Survey (NHANES) database, encompassing the years 1999 through to 2018. The NHANES is a continual survey employing a comprehensive, multi-stage probability sampling methodology to select a representative sample of the U.S. population, with a primary focus on assessing the health and nutritional status of American adults and children. The NHANES research protocol has secured approval from the Institutional Review Board of the National Center for Health Statistics (NCHS), and all study participants provided written informed consent. More in-depth information regarding this can be accessed at www.cdc.gov/nchs/nhanes/irba98.htm.

**Figure 1:**
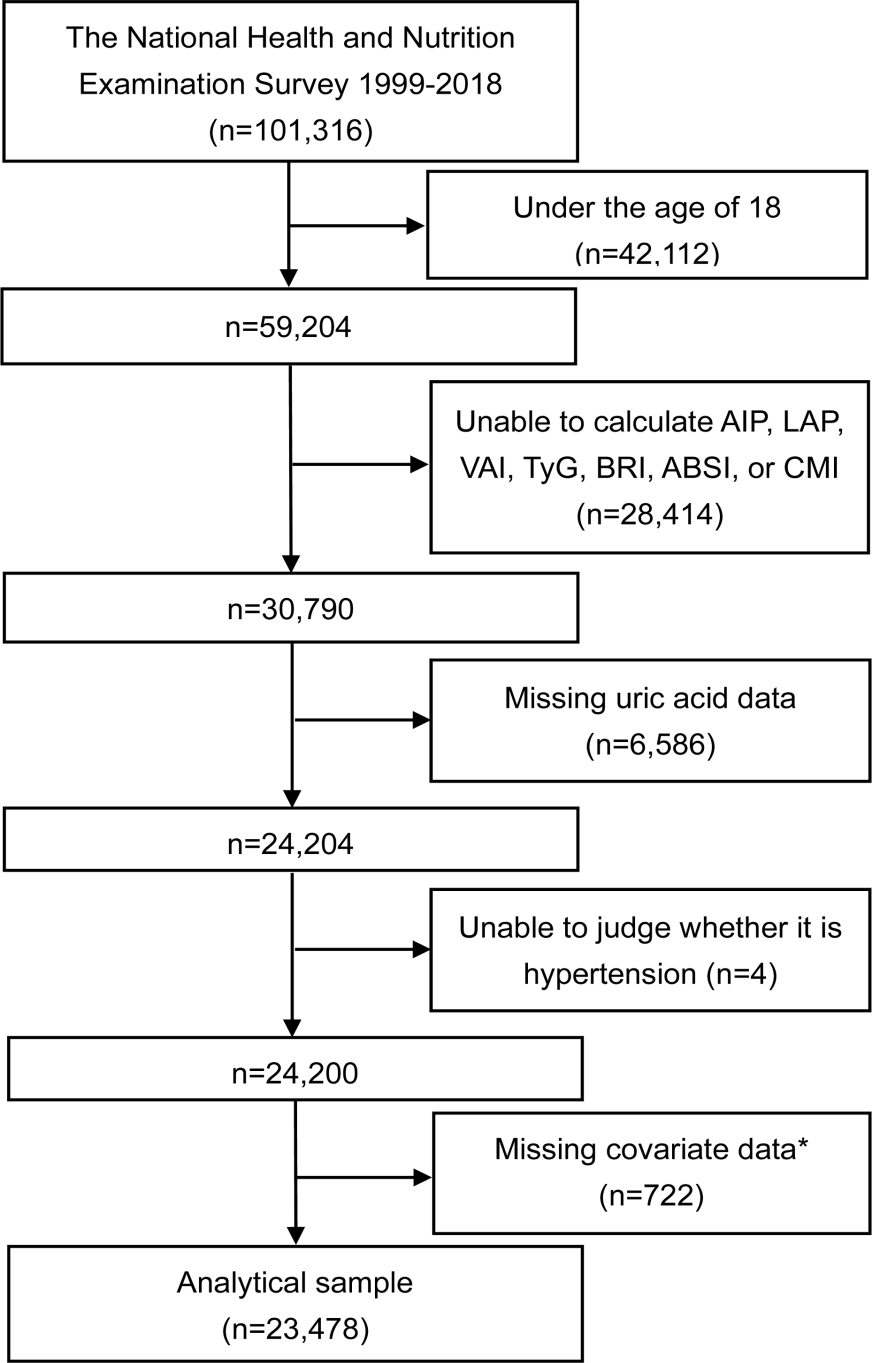
Flowchart of the study. *The covariates include age, gender, race/ethnicity, education level, PIR, smoking, alcohol consumption, MET, SBP, DBP, FPG, HbA1c, creatinine, urea nitrogen, TC, LDL-C, eGFR, hypoglycemic drugs, and lipid-lowering drugs. AIP, atherogenic index of plasma; LAP, lipid accumulation product; VAI, visceral adiposity index; TyG, triglyceride-glucose index; BRI, body roundness index; ABSI, a body shape index; CMI, cardiometabolic index; PIR, poverty income ratio; MET, metabolic equivalent of task; SBP, systolic blood pressure; DBP, diastolic blood pressure; FPG, fasting plasma glucose; HbA1c, glycated hemoglobin; TC, total cholesterol; LDL-C, low-density lipoprotein cholesterol; eGFR, estimated glomerular filtration rate.

This study utilizes data from the NHANES database, collected from 1999 to 2018, initially comprising 101,316 participants. Subjects were excluded under the following conditions: aged under 18 years (n=42,112), inability to calculate AIP, LAP, VAI, TyG, BRI, ABSI, or CMI (missing data on total triglyceride (TG), high-density lipoprotein cholesterol (HDL-C), waist circumference (WC), BMI, or fasting plasma glucose (FPG)) (n=28,414), missing uric acid values (n=6,586), inability to diagnose HTN (n=4), and missing covariates (n=722). Following these exclusions, the analysis includes 23,478 participants with complete data sets, as shown in **Figure 1**.

### 2.2 Definitions of seven anthropometric indexes

In this study, anthropometric indexes included AIP, LAP, VAI, TyG, BRI, ABSI, and CMI. The AIP was calculated using the formula (20):


$$\text{AIP }=\text{ log}\left(\frac{\text{TG(mg/dL)}}{\text{HDL}-\text{C}(\text{mg}/\text{dL})}\right)$$

The LAP was calculated as follows (21):


Males: LAP = (WC(cm)−65)×TG(mmol/L)

Female:LAP=(WC(cm)− 58)×TG(mmol/L)

The VAI was determined by the formula (22):


$$\text{Males: VAI=}\frac{\text{WC(cm)}}{39.68+\left(1.88\times {\text{BMI(kg/m}}^{2})\right)}\;\times \left(\frac{\text{TG(mmol/L}}{1.03}\right)\times \left(\frac{1.31}{\text{HDL}-\text{C(mmol/L)}}\right)\;$$

$$\text{Females: VAI }=\;\frac{\text{WC(cm)}}{36.58+\left(1.89\times {\text{BMI(kg/m}}^{2})\right)}\times \left(\frac{\text{TG(mmol/L}}{0.81}\right)\;\times \;\left(\frac{1.52}{\text{HDL}-\text{C}(\text{mmol}/\text{L})}\right)$$

The formula for calculating the TyG index is as follows (23):


$$\text{TyG }=\;1\text{n }\left(\frac{\text{TG(mg/dL)}\times \text{FPG(mg/dL)}}{2}\right)$$

The BRI was calculated using the following formula (24):


$$\text{BRI }=\;364.2-365.5\times \sqrt{1-\left(\frac{{\text{WC(cm)/(2π)}}^{2}}{{(0.5\times \text{height(cm))}}^{2}}\right)}$$

The ABSI was based on WC adjusted for height and weight (25):


$$\text{ABSI = }\frac{\text{WC(m)}}{{\text{BMI(kg/m}}^{2}{)}^{\frac{2}{3}}\times {\text{height(m)}}^{\frac{1}{2}}}$$

The CMI was calculated using the formula (26):


$$\text{CMI }=\frac{\text{TG}(\text{mmol}/L)}{\text{HDL}-\text{C}(\text{mmol}/\text{L})}\times \frac{\text{WC}(\text{cm})}{\text{height(cm)}}$$

### 2.3 Assessment of the diagnosis of HTN and HUA

HTN was defined as s average blood pressure ≥ 140/90 mmHg, a history of HTN and/or the use of antihypertensive drugs in health questionnaire. The average blood pressure is determined using the following protocol: (1) Any diastolic reading of zero is not included in the calculation of the diastolic average; (2) If all diastolic readings are zero, the average is set as zero; (3) If only one blood pressure reading is available, it is taken as the average; (4) If multiple blood pressure readings are available, the first reading is excluded from the average calculation.

Adhering to established diagnostic criteria, HUA was defined as serum uric acid levels exceeding a threshold of 7.0 mg/dL in males and 6.0 mg/dL in females (30). The serum uric acid level was assessed using either the Beckman UniCel^®^ DxC800 Synchron or the Beckman Synchron LX20 (Beckman Coulter, Inc., Brea, CA, United States). These systems utilize an oxidation process that converts uric acid to allantoin and H_2_O_2_.

### 2.4 Covariates

This study utilized a computer-assisted personal interview to gather data on demographic and lifestyle variables, physical measurements, and laboratory test results. Demographic data included age, sex, race/ethnicity, educational level, and poverty income ratio (PIR). The latter was computed by dividing the family income by the poverty threshold, and was categorized into three levels:<1.3 (low income), 1.3–3.5 (moderate income), and >3.5 (high income). Health status assessment covered smoking and drinking habits, physical activity, and medication history (antidiabetic and lipid-lowering medications). Smoking status was divided into three categories: never smokers (smoked less than 100 cigarettes in their lifetime), former smokers (smoked over 100 cigarettes but quit at the time of the survey), and current smokers (smoked over 100 cigarettes and continue to smoke). Alcohol consumption was also classified into three levels: heavy drinking (females: ≥3 drinks/day or binge drinking on 5+ days/month; males: ≥4 drinks/day or same binge drinking frequency), moderate drinking (females: ≥2 drinks/day or binge drinking ≥2 days/month; males: ≥3 drinks/day or same binge drinking frequency), and mild drinking (others). Physical activity was evaluated using the metabolic equivalent of task (MET)/week, a measure calculated by multiplying the total minutes spent on various activities during the week by their respective metabolic equivalents (Compendium of Physical Activities). The physical activity level was divided into three groups: low (<600 METs/week), moderate (600-1199 METs/week), and vigorous (≥1200 METs/week). The physical health examination included measurements of blood pressure, while laboratory tests were conducted to measure FPG and estimate the glomerular filtration rate (eGFR). The eGFR was computed using the 2009 Serum Creatinine (SCr)-based Chronic Kidney Disease Epidemiology Collaboration (CKD-EPI) equation (31).

### 2.5 Statistical analysis

In this study, baseline characteristics were reported as means and standard deviations (SD) for continuous variables, and as proportions for categorical variables. Student’s t-test or the chi-square test were employed for the analysis of normally distributed variables. For variables with skewed distributions, non-parametric tests or Fisher’s exact probability tests were utilized. To explore the association between various anthropometric indexes and HUA, HTN, and HTN-HUA, multivariate logistic regression analyses were performed. Receiver operating characteristic (ROC) curve analyses, along with the area under the curve (AUC), were then employed to evaluate the discriminative ability of the AIP, LAP, VAI, TyG, BRI, ABSI, and CMI in relation to HUA, HTN, and HTN-HUA. The Youden index was used to determine the cut-off values for these indexes by identifying the highest value on the ROC curves. In addition, decision curve analysis (DCA) was used to calculate the net benefit for each risk threshold probability to compare the clinical value of the seven anthropometric indicators. This approach helps in understanding the practical implications of using these indexes in a clinical setting by quantifying their net benefits at various threshold probabilities. The DeLong’s test for statistical significance was used to test differences between AUC curves (32). Moreover, bootstrap resampling (conducted 500 times) served as a sensitivity analysis in the assessment of AUC to verify the stability of the results. Statistical analyses were conducted using R (version 3.5.3) and EmpowerStats (http://www.EmpowerStats.com). A P-value of less than 0.05 was considered statistically significant.

## 3 Results

### 3.1 Baseline characteristics

**Table 1** presents the baseline characteristics of 23,478 study participants, which included 2,015 (8.58%) with HTN alone, 6,537 (27.84%) with HUA alone, and 2,836 (12.08%) with HTN-HUA. Comparatively, the HTN-HUA group differed significantly from the control group across all variables, with the exception of age and PIR. This group was generally older, with a higher proportion having high school education or less. They also had a higher incidence of former smoking and drinking. Notably, the HTN-HUA group demonstrated a lower METs/week, higher BMI, larger WC, and higher blood pressure. This group also showed elevated levels of FPG, uric acid, and TG, alongside lower HDL-C, eGFR, and a higher proportion of antidiabetic and lipid-lowering medications (p< 0.05). In addition, the only exceptions in anthropometric indexes were AIP between HUA alone group and HTN-HUA group and BRI between HTN alone group and HUA alone group - these showed no statistical differences. All other anthropometric indexes revealed significant differences (p< 0.05). It is important to note that the HTN-HUA group exhibited higher anthropometric indexes than the other groups (p< 0.05).

**Table 1:** Baseline characteristics of subjects.

Variables	C n=12090	HTN alone n=6537	HUA alone n=2015	HTN-HUA n=2836
**Age (years)**	40.06 ± 16.77	58.80 ± 15.81	41.41 ± 18.24	60.69 ± 15.40
Sex, n (%)				
Male	5777 (47.78%)^a^	3270 (50.02%)^c^	1329 (65.96%)	1393 (49.12%)^a,c^
Female	6313 (52.22%)^a^	3267 (49.98%)^c^	686 (34.04%)	1443 (50.88%)^a,c^
Race/ethnicity, n (%)				
Non-Hispanic White	5022 (41.54%)	2943 (45.02%)	910 (45.16%)	1357 (47.85%)
Non-Hispanic Black	2163 (17.89%)	1498 (22.92%)	359 (17.82%)	792 (27.93%)
Mexican American	2620 (21.67%)	1070 (16.37%)	361 (17.92%)	287 (10.12%)
Others	2285 (18.90%)	1026 (15.70%)	385 (19.11%)	400 (14.10%)
Education level, n (%)				
Less than high school	3239 (26.81%)^a^	2044 (31.31%)	494 (24.55%)^a^	798 (28.18%)
High school	2789 (23.09%)^a^	1584 (24.26%)	482 (23.96%)^a^	738 (26.06%)
More than high school	6052 (50.10%)^a^	2901 (44.43%)	1036 (51.49%)^a^	1296 (45.76%)
PIR, n (%)				
Low	3529 (31.97%)^a^	1845 (31.09%)^c^	515 (27.87%)	791 (30.41%)^a,c^
Medium	4122 (37.34%)^a^	2347 (39.55%)^c^	708 (38.31%)	1033 (39.72%)^a,c^
High	3388 (30.69%)^a^	1742 (29.36%)^c^	625 (33.82%)	777 (29.87%)^a,c^
Smoking, n (%)				
Never	6360 (52.61%)	3249 (49.70%)	996 (49.43%)	1366 (48.17%)
Former	2147 (17.76%)	1952 (29.86%)	461 (22.88%)	989 (34.87%)
Now	2518 (20.83%)	1269 (19.41%)	387 (19.21%)	459 (16.18%)
Not reported	1065 (8.81%)	67 (1.02%)	171 (8.49%)	22 (0.78%)
Drinking, n (%)				
Never	1403 (11.60%)	927 (14.18%)^c^	187 (9.28%)	403 (14.21%)^c^
Former	1347 (11.14%)	1330 (20.35%)^c^	230 (11.41%)	612 (21.58%)^c^
Mild	3357 (27.77%)	2115 (32.35%)^c^	538 (26.70%)	864 (30.47%)^c^
Moderate	1711 (14.15%)	713 (10.91%)^c^	266 (13.20%)	304 (10.72%)^c^
Heavy	2304 (19.06%)	872 (13.34%)^c^	497 (24.67%)	408 (14.39%)^c^
Not reported	1968 (16.28%)	580 (8.87%)^c^	297 (14.74%)	245 (8.64%)^c^
METs/week, n (%)				
Low	3274 (27.08%)^a^	1636 (25.03%)	537 (26.65%)^a^	701 (24.72%)
Moderate	1381 (11.42%)^a^	696 (10.65%)	223 (11.07%)^a^	304 (10.72%)
Vigorous	4897 (40.50%)^a^	2152 (32.92%)	814 (40.40%)^a^	850 (29.97%)
Not reported	2538 (20.99%)^a^	2053 (31.41%)	441 (21.89%)^a^	981 (34.59%)
**BMI (kg/m^2^)**	26.78 ± 5.74	29.53 ± 6.46	31.05 ± 7.05	32.59 ± 7.51
**WC (cm)**	92.14 ± 14.47	101.61 ± 15.11	103.76 ± 16.43	109.02 ± 16.00
**SBP (mmHg)**	114.12 ± 11.00	136.62 ± 20.36	117.88 ± 10.43	135.55 ± 21.47
**DBP (mmHg)**	67.49 ± 9.75	73.01 ± 13.89	69.79 ± 10.46	71.84 ± 14.56
**FPG (mg/dL)**	101.17 ± 29.29	116.67 ± 44.80	103.38 ± 21.46	117.67 ± 38.51
**Uric acid (mg/dL)**	4.90 ± 1.02	5.11 ± 0.98	7.37 ± 0.89	7.52 ± 1.10
**TG (mg/dL)**	113.46 ± 99.75	137.88 ± 112.76	157.16 ± 172.96	164.29 ± 129.63
**HDL-C (mg/dL)**	54.22 ± 15.23^a^	54.37 ± 16.73^a^	47.77 ± 14.07	49.95 ± 15.32
**eGFR (ml/min/1.73 m^2^)**	104.87 ± 20.19	87.88 ± 22.20	97.34 ± 23.70	75.61 ± 25.25
**Antidiabetic medications, n (%)**	494 (4.09%)	1167 (17.85%)	107 (5.31%)	614 (21.65%)
**Lipid lowering medications (n, %)**	841 (6.96%)	2033 (31.10%)	175 (8.68%)	974 (34.34%)
**AIP**	-0.10 ± 0.32	-0.01 ± 0.33	0.09 ± 0.33^d^	0.10 ± 0.32^d^
**LAP**	42.66 ± 51.87	64.67 ± 62.66	74.63 ± 85.73	88.92 ± 76.76
**VAI**	1.75 ± 2.32	2.23 ± 2.97	2.60 ± 3.48	2.89 ± 3.26
**TyG**	8.45 ± 0.65	8.77 ± 0.69	8.78 ± 0.64	8.97 ± 0.65
**BRI**	4.51 ± 1.95	5.83 ± 2.15^b^	5.83 ± 2.42^b^	6.89 ± 2.48
**ABSI**	0.0799 ± 0.0049	0.0829 ± 0.0049	0.0809 ± 0.0047	0.0834 ± 0.0049
**CMI**	1.43 ± 2.01	1.92 ± 2.61	2.39 ± 3.41	2.57 ± 3.06

Groups that share the same superscript letter do not exhibit any statistical difference between them. Conversely, a superscript with no letter indicates that the group is statistically different from all other groups.

HTN, hypertension; HUA, hyperuricemia; HTN-HUA, hypertension plus hyperuricemia; PIR, poverty income ratio; MET, metabolic equivalent of task; BMI, body mass index; WC, waist circumference; SBP, systolic blood pressure; DBP, diastolic blood pressure; FPG, fasting plasma glucose; TG, total triglyceride; HDL-C, high-density lipoprotein cholesterol; eGFR, estimated glomerular filtration rate; AIP, atherogenic index of plasma; LAP, lipid accumulation product; VAI, visceral adiposity index; TyG, triglyceride-glucose index; BRI, body roundness index; ABSI, a body shape index; CMI, cardiometabolic index.

**Figure 2:**
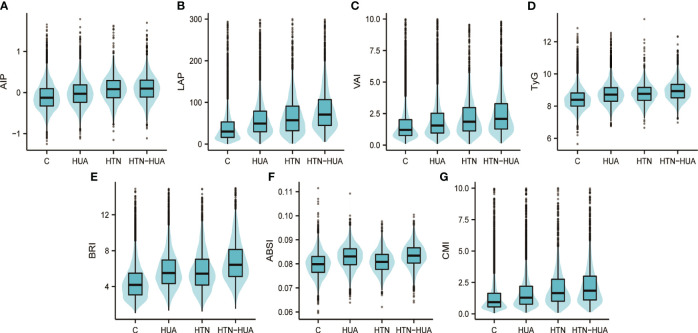
Atherogenic index of plasma **(A)**, lipid accumulation product **(B)**, visceral adiposity index **(C)**, triglyceride-glucose index **(D)**, body roundness index **(E)**, a body shape index **(F)**, cardiometabolic index **(G)** values in different groups. Group C refers to participants who have neither hypertension nor hyperuricemia. HTN, hypertension alone; HUA, hyperuricemia alone; HTN-HUA, hypertension plus hyperuricemia; AIP, atherogenic index of plasma; LAP, lipid accumulation product; VAI, visceral adiposity index; TyG, triglyceride-glucose index; BRI, body roundness index; ABSI, a body shape index; CMI, cardiometabolic index.

**Figure 2** visualizes the differences in the seven anthropometric indexes - AIP, LAP, VAI, TyG, BRI, ABSI, and CMI - among the different groups. Notably, all these indexes were significantly higher in the HTN-HUA group compared to the other three groups.

### 3.2 Association between seven anthropometric indexes and risks of HUA alone, HTN alone and HTN-HUA

**Table 2** presents the effect sizes of seven anthropometric indexes (AIP, LAP, VAI, TyG, BRI, ABSI, and CMI) and their association with the risks of HUA alone, HTN alone, and HTN-HUA. After adjusting for variables such as age, gender, race/ethnicity, education level, PIR, smoking, alcohol consumption, MET, eGFR, and use of antidiabetic and lipid-lowering medications, each anthropometric index showed a significant association with all three conditions (p< 0.05). However, only AIP was found to have no significant association with HTN alone (p > 0.05). Among the three groups, all the anthropometric indexes demonstrated the highest ORs for HTN-HUA. Specifically, the ORs of the highest quartile of the seven indexes for HTN-HUA were as follows: AIP had an OR of 4.45 (95% CI 3.82-5.18), LAP an OR of 9.52 (95% CI 7.82-11.59), VAI an OR of 4.53 (95% CI 3.89-5.28), TyG an OR of 4.91 (95% CI 4.15-5.80), BRI an OR of 9.08 (95%CI 7.45-11.07), ABSI an OR of 1.71 (95%CI 1.45-2.02), and CMI an OR of 6.57 (95%CI 5.56-7.76).

**Table 2:** Odd ratios^*^ and 95% confidence intervals for highest versus the lowest quartiles in logistic regressions predicting presence of HTN alone, HUA alone and HTN-HUA.

	HTN alone	HUA alone	HTN-HUA
**AIP**	1.06 (0.96, 1.17)	3.69 (3.13, 4.35)	4.45 (3.82, 5.18)
**LAP**	1.47 (1.33, 1.63)	5.76 (4.88, 6.80)	9.52 (7.82, 11.59)
**VAI**	1.11 (1.00, 1.22)	3.65 (3.12, 4.26)	4.53 (3.89, 5.28)
**TyG**	1.21 (1.09, 1.34)	3.45 (2.93, 4.06)	4.91 (4.15, 5.80)
**BRI**	1.69 (1.52, 1.88)	4.44 (3.79, 5.21)	9.08 (7.45, 11.07)
**ABSI**	1.13 (1.01, 1.26)	1.64 (1.37, 1.95)	1.71 (1.45, 2.02)
**CMI**	1.14 (1.03, 1.26)	4.36 (3.70, 5.14)	6.57 (5.56, 7.76)

Adjusted for age, sex, race/ethnicity, education level, PIR, smoking, drinking, MET, eGFR, antidiabetic medication, and lipid-lowering medication.

HTN, hypertension; HUA, hyperuricemia; HTN-HUA, hypertension plus hyperuricemia; AIP, atherogenic index of plasma; LAP, lipid accumulation product; VAI, visceral adiposity index; TyG, triglyceride-glucose index; BRI, body roundness index; ABSI, a body shape index; CMI, cardiometabolic index; PIR, poverty income ratio; MET, metabolic equivalent of task; eGFR, estimated glomerular filtration rate.

Sensitivity analysis using a serum uric acid threshold of 6.5 mg/dL yielded similar results to those in **Table 2**, with the notable difference being the lack of a statistically significant association between ABSI and HTN alone, except for the non-association of AIP with normouricemia in hypertensive patients (**Supplementary Table 1**).

### 3.3 AUCs and cut-off values of seven anthropometric indexes for prediction of HUA alone, HTN alone and HTN-HUA

**Figure 3:**
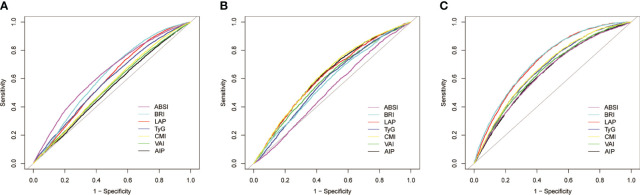
Receiver operating characteristic curves of seven anthropometric indexes for prediction of **(A)** hypertension alone; **(B)** hyperuricemia alone; and **(C)** hypertension plus hyperuricemia. ABSI, a body shape index; BRI, body roundness index; LAP, lipid accumulation product; TyG, triglyceride-glucose index; CMI, cardiometabolic index; VAI, visceral adiposity index; AIP, atherogenic index of plasma.

**Table 3** and **Figure 3** show the AUC values of AIP, LAP, VAI, TyG, BRI, ABSI, and CMI for discriminating HUA alone, HTN alone, and HTN-HUA. All the anthropometric indexes demonstrated the highest AUCs for HTN-HUA among the three groups. Specifically, LAP and BRI exhibited significant discriminative ability for HTN-HUA, with AUC values of 0.72 (95% CI 0.71–0.73) and 0.73 (95% CI 0.72–0.74), respectively. To discriminate the patients with HTN-HUA, the cut-off value for LAP was 43.32, and for BRI it was 5.23. The DeLong test, which was employed to evaluate the differences in the predictive ability of HTN-HUA between the four indexes, revealed no statistically significant difference between the AUC of LAP and BRI (p > 0.13) (**Supplementary Table 2**).

**Table 3:** Area under the curve and cut off values of seven anthropometric indexes for prediction of HTN alone, HUA alone and HTN-HUA.

	AUC	95%CI low	95%CI upp	Cut off Value	Specificity	Sensitivity
HTN alone						
AIP	0.53	0.52	0.54	-0.17	0.37	0.68
LAP	0.59	0.58	0.60	31.60	0.42	0.73
VAI	0.54	0.53	0.55	1.02	0.34	0.73
TyG	0.58	0.57	0.59	8.57	0.53	0.59
BRI	0.63	0.61	0.63	4.53	0.47	0.72
ABSI	0.64	0.63	0.64	0.08	0.65	0.55
CMI	0.55	0.54	0.56	0.92	0.41	0.67
HUA alone						
AIP	0.62	0.61	0.63	-0.01	0.56	0.63
LAP	0.62	0.61	0.63	43.90	0.54	0.65
VAI	0.60	0.59	0.61	1.46	0.52	0.64
TyG	0.58	0.57	0.60	8.58	0.52	0.63
BRI	0.58	0.57	0.59	4.72	0.47	0.65
ABSI	0.52	0.51	0.54	0.08	0.26	0.79
CMI	0.63	0.62	0.64	1.10	0.49	0.71
HTN-HUA						
AIP	0.64	0.63	0.65	-0.01	0.58	0.63
LAP	0.72	0.71	0.73	43.32	0.56	0.77
VAI	0.65	0.64	0.66	1.61	0.58	0.65
TyG	0.67	0.66	0.68	8.63	0.57	0.68
BRI	0.73	0.72	0.74	5.23	0.60	0.73
ABSI	0.64	0.63	0.65	0.08	0.54	0.67
CMI	0.67	0.66	0.68	1.17	0.53	0.73

AUC, area under the curve; CI, confidence interval; HTN, hypertension; HUA, hyperuricemia; HTN-HUA, hypertension plus hyperuricemia; AIP, atherogenic index of plasma; LAP, lipid accumulation product; VAI, visceral adiposity index; TyG, triglyceride-glucose index; BRI, body roundness index; ABSI, a body shape index; CMI, cardiometabolic index.

Additionally, sensitivity analysis with serum uric acid set at 6.5 mg/dL as the threshold yielded similar results. LAP and BRI were the most effective in discriminating HTN-HUA, followed by CMI, TyG, ABSI, AIP, and VAI (**Supplementary Table 3**). However, in the DCA analysis we can see that BRI has the largest net clinical benefit (**Supplementary Figure 1**).

We also provided AUCs and cut-off values of the seven anthropometric indexes for predicting HUA alone, HTN alone, and HTN-HUA, both stratified by sex (**Supplementary Tables 4**–**6**, and **Supplementary Figures 2**, **3**) and analyzed using bootstrap resampling (times = 500) (**Supplementary Table 7**, and **Supplementary Figures 4**–**6**). Similarly, we verified the stability of the above results in stratified analysis and bootstrap resampling analysis as sensitivity analyses.

## 4 Discussion

In this cross-sectional study, utilizing data from NHANES, we discovered that the prevalence of HTN was at 39.92%, with patients with HTN-HUA constituting 21.50% of the hypertensive population. Numerous studies have indicated that elevated blood uric acid levels increase the risk of cardiovascular events in hypertensive patients (11–13). As such, the early detection and management of HTN-HUA through anthropometric indexes, prior to the onset of clinical symptoms, could be crucial in managing HTN-HUA and preventing associated cardiovascular events.

In light of recent findings from the Uric Acid Right for Heart Health (URRAH) study, particularly those published in Maloberti et al. (33), reconsideration of the established diagnostic criteria for HUA in the context of cardiovascular risk is warranted. While our study adheres to the conventional threshold of 7.0 mg/dL in males and 6.0 mg/dL in females (30), primarily associated with gout implications, the URRAH research suggests a significantly lower cut-off of 6.5 mg/dL for both sexes concerning cardiovascular mortality. This insight is crucial for our study’s scope, which focuses on anthropometric indexes in predicting HTN-HUA among U.S. adults. Integrating this nuanced understanding of uric acid levels in relation to cardiovascular risk, possibly as sensitivity analyses, would enhance the depth of our analysis. Another study from Italy revealed that traditional HUA cut-offs are associated with higher ORs for obesity indices compared to the URRAH thresholds, with the LAP demonstrating the most significant association with HUA (34). These observations align with our findings, suggesting a multifaceted interplay between uric acid, lipids, and obesity in the general population. It appears that lower serum uric acid levels primarily impact cardiovascular events through lipid modifications, whereas higher serum uric acid levels may further precipitate metabolic and obesity-related abnormalities. Future studies are essential to further analyze and validate these complex relationships.

To investigate the relationship between obesity and HTN-HUA, we utilized anthropometric indexes, which are measured by simple variables such as sex, TG, HDL-C, WC, BMI, and FPG. In this study, seven such anthropometric indexes were found to have significant associations with HTN-HUA. The odds ratios were especially high for LAP and BRI. LAP, calculated mainly based on sex, TG, and WC, had been previously employed to gauge the extent of lipid accumulation in the body (21). The VAI focuses more on the extent of visceral fat accumulation (22), as compared to the LAP with a higher overlap of calculated variables. Liu et al. found that high VAI is a measure of visceral fat and metabolic dysfunction, and is an independent risk factor for HUA in hypertensive people (35). However, Li et al. found that LAP was a better predictor of metabolic syndrome than VAI in both genders (36). This indicates that the overall lipid accumulation in the body, rather than solely visceral fat accumulation, may better predict HTN-HUA and other metabolic disorders. For example, Neeland et al. discovered a strong association between ectopic fat and the onset of clinical syndromes characterized by atherosclerotic dyslipidemia, hyperinsulinemia/glucose intolerance, HTN, atherosclerosis, and adverse cardiac remodeling/heart failure (37). Concurrently, research has shown that despite visceral fat being more closely linked to poor metabolic risk status, subcutaneous fat still contributes to unfavorable metabolic outcomes (38).

The BRI calculation primarily involves WC and height, and is predominantly used to evaluate obesity distribution in humans (24). The ABSI calculation incorporates several BRI variables as well as BMI, another measure used to assess human obesity distribution (25). However, there’s a distinction between the two: BRI is more commonly used to evaluate an individual’s overall physical fitness, while ABSI is more targeted toward reflecting the health implications of abdominal obesity. A study by Anto et al. revealed that after adjusting for all variables, the odds ratio of ABSI on the risk of metabolic syndrome was not statistically significant (p > 0.05), while BRI remained significant (p< 0.05) (39). Similarly, when identifying metabolic disorders in both adult and pediatric populations in China, BRI was found to possess superior predictive power compared to ABSI (40, 41). All of this suggests a higher predictive value of BRI than ABSI in forecasting metabolic disorders. Nevertheless, a study from China reported a significant non-linear positive dose-response relationship between all anthropometric measures, except ABSI, and HTN across sexes (p-nonlinearity< 0.05), including BRI (42). This study, however, was limited to a target population aged over 65 years. It is well-documented that age is a significant risk factor for HTN, with its prevalence increasing as people age (43–45). Consequently, the outcomes of the non-linear analysis may not be generalizable to the adult population in the U.S.

The TyG, a simple surrogate marker of insulin resistance, is calculated using TG and FPG (23). It’s notable that obese individuals often exhibit insulin resistance and lipoprotein metabolism disorders, such as heightened plasma concentrations of triglyceride-rich lipoprotein residues, residue-like particulate cholesterol, and apolipoprotein B, all of which are more pronounced in obese individuals with hypertriglyceridemia (46). Furthermore, elevated TG levels in obese individuals are reported to be linked to insulin resistance, underscoring the significance of TG in the pathogenesis of insulin resistance (47). However, some studies suggest that due to the crucial role of obesity in the pathophysiology of insulin resistance, integrating obesity markers with TyG for predicting metabolic disorders in humans could yield superior results (48–50). Therefore, relying solely on fasting TG and FPG may not be sufficient, and a better strategy might be to combine these with indexes that directly measure obesity in humans.

The AIP, derived from TG and HDL-C, has been correlated with insulin resistance and abnormalities in lipid metabolism (51, 52). Tan et al. discovered that an elevated AIP is significantly and positively associated with the risk of developing prehypertension or HTN in normoglycemic individuals, particularly in women aged 40 to 60 (53). Conversely, Li et al. found a stronger correlation between AIP and HTN risk in men (54). This observation might stem from the fact that individuals with prehypertension or HTN often exhibit chronic abnormalities in serum concentrations of TG, cholesterol, or both, as well as in associated lipoproteins (55). However, in this study, AIP demonstrated the least efficacy in discriminating HTN-HUA among the seven anthropometric measures assessed, and a multivariate-adjusted logistic regression with HTN alone did not yield any statistical significance. This discrepancy could be attributed to the interplay of regional and ethnic differences, lifestyle habits, and other variables (56), resulting in variations between the findings of the current study and previous research. Concurrently, several studies have illustrated that HUA can modulate molecular signals such as insulin resistance, inflammatory response, oxidative stress, endoplasmic reticulum stress, and endothelial dysfunction (57, 58). This modulation might explain the absence of observed true associations in cases of HTN alone.

The CMI is a relatively new index associated with lipid and obesity (26). Differing from AIP, CMI is calculated not only based on TG and HDL-C but also incorporates WC and height. Numerous studies have attested to the positive correlation between CMI and various metabolic disorders (26, 59, 60). In this study, CMI demonstrated moderate predictive power for HTN-HUA but did not exhibit stronger predictive power. From the perspective of anthropometric index components, the calculation of CMI encompasses the components of both AIP and BRI. The findings of this study could possibly suggest some degree of collinearity between the calculated components of the CMI. Firstly, a significant correlation between WC and lipids is well established (61). Secondly, TG and HDL-C, which are crucial components of lipids, may not enhance the predictive power for HTN-HUA when combined. This was also supported by the prediction of HTN-HUA by AIP in this study.

This study offers both strengths and limitations. Being the first large-scale study to examine the relationship between anthropometric indexes and HTN-HUA in an adult population using a nationally representative sample, it adds statistical strength and verifies the reliability of the reported results. However, several limitations warrant attention. Firstly, the study does not adequately establish the causal relationship between these anthropometric indexes and HTN-HUA, and future longitudinal studies are needed to verify this causal relationship. Secondly, the use of retrospective data in our study may introduce recall bias. Thirdly, there may be probability bias in this study, as the study population consists solely of individuals from the United States, the conclusions drawn may not be universally applicable. Fourthly, the lack of data on hypouricemic drugs, diuretics, and Sodium-Glucose Co-Transporter 2 (SGLT2) inhibitors in the current survey may influence uric acid levels (62, 63), which may have affected the results of the analysis in this study. Lastly, the significant absence of inflammatory markers like high-sensitivity C-reactive protein in our data restricts their inclusion in the regression model as adjusting variables. This omission affects the interpretation of the internal health dynamics in individuals with HTN-HUA (64), an aspect that warrants attention in future research endeavors.

## 5 Conclusion

In conclusion, this study underscores that various indexes, including AIP, LAP, VAI, TyG, BRI, ABSI, and CMI, are closely associated with HTN-HUA risk, often more so than HTN or HUA alone. Among these, LAP and BRI emerge as particularly noteworthy due to their pronounced ability to discriminate HTN-HUA risk. However, it is important to highlight that while both LAP and BRI are statistically robust indexes for predicting HTN-HUA, BRI stands out as more effective. The primary advantage of BRI lies in its non-invasive nature, eliminating the need for invasive testing procedures. This makes BRI not only a powerful tool in risk assessment but also a more practical and patient-friendly option in clinical settings. Consequently, BRI’s accessibility and efficacy position it as a superior choice for early warning indexes in managing HTN-HUA. Additionally, its non-invasive character enhances its suitability for use in obesity-based prevention and intervention strategies for HTN-HUA, broadening its applicability in public health initiatives.

## Data availability statement

The raw data supporting the conclusions of this article will be made available by the authors, without undue reservation.

## Ethics statement

All data came from NHANES, which was approved by National Centre for Health Statistics Institutional Ethics Review Board, and all the subjects agreed on the survey and signed written consent. The studies were conducted in accordance with the local legislation and institutional requirements. The participants provided their written informed consent to participate in this study.

## Author contributions

YL: Data curation, Formal analysis, Investigation, Methodology, Validation, Visualization, Writing – original draft. LZ: Conceptualization, Funding acquisition, Project administration, Resources, Software, Supervision, Validation, Visualization, Writing – review & editing.
