# Supplementary material for: Comparison of seven anthropometric indexes to predict hypertension plus hyperuricemia among U.S. adults
Source: Front Endocrinol (Lausanne). 2024 Mar 8;15:1301543. doi: 10.3389/fendo.2024.1301543 (PMC10958198; doi:10.3389/fendo.2024.1301543)
Supplement: Supplementary file 1 [file DataSheet_1.docx]

**Table S1. Odd ratios^*^ and 95% confidence intervals for highest versus the lowest quartiles in logistic regressions predicting presence of** **HTN alone, HUA alone and HTN-HUA (6.5 mg/dL as threshold for serum uric acid)**

|  | **HTN alone** | **HUA alone** | **HTN-HUA** |
| --- | --- | --- | --- |
| **AIP** | 1.09 (0.98, 1.21) | 3.20 (2.75, 3.74) | 4.05 (3.48, 4.70) |
| **LAP** | 1.52 (1.36, 1.69) | 3.97 (3.42, 4.61) | 8.22 (6.84, 9.88) |
| **VAI** | 1.15 (1.04, 1.27) | 2.88 (2.50, 3.31) | 3.88 (3.36, 4.49) |
| **TyG** | 1.26 (1.14, 1.40) | 2.74 (2.36, 3.19) | 4.36 (3.71, 5.13) |
| **BRI** | 1.71 (1.53, 1.90) | 3.02 (2.61, 3.51) | 8.63 (7.17, 10.39) |
| **ABSI** | 1.06 (0.95, 1.19) | 1.60 (1.35, 1.90) | 2.06 (1.73, 2.44) |
| **CMI** | 1.21 (1.09, 1.34) | 3.68 (3.16, 4.29) | 5.45 (4.64, 6.41) |

**Notes:** Adjusted for age, sex, race/ethnicity, education level, PIR, smoking, drinking, MET, eGFR, antidiabetic medication, and lipid-lowering medication.

**Abbreviations:** HTN, hypertension; HUA, hyperuricemia; HTN-HUA, hypertension plus hyperuricemia; AIP, atherogenic index of plasma; LAP, lipid accumulation product; VAI, visceral adiposity index; TyG, triglyceride-glucose index; BRI, body roundness index; ABSI, a body shape index; CMI, cardiometabolic index; PIR, poverty income ratio; MET, metabolic equivalent of task; eGFR, estimated glomerular filtration rate.

**Table S2. Comparison of area under the curves for seven anthropometric indexes using DeLong's test**

|  | **AIP** | **LAP** | **VAI** | **TyG** | **BRI** | **ABSI** | **CMI** |
| --- | --- | --- | --- | --- | --- | --- | --- |
| **AIP** | / | <0.05 | <0.05 | <0.05 | <0.05 | 0.95 | <0.05 |
| **LAP** | / | / | <0.05 | <0.05 | 0.13 | <0.05 | <0.05 |
| **VAI** | / | / | / | <0.05 | <0.05 | 0.05 | <0.05 |
| **TyG** | / | / | / | / | <0.05 | <0.05 | 0.12 |
| **BRI** | / | / | / | / | / | <0.05 | <0.05 |
| **ABSI** | / | / | / | / | / | / | <0.05 |
| **CMI** | / | / | / | / | / | / | / |

**Abbreviations:** AIP, atherogenic index of plasma; LAP, lipid accumulation product; VAI, visceral adiposity index; TyG, triglyceride-glucose index; BRI, body roundness index; ABSI, a body shape index; CMI, cardiometabolic index.

**Table S3. Area under the curve and cut off values of seven anthropometric indexes for prediction of HTN alone, HUA alone and HTN-HUA (6.5 mg/dL as threshold for serum uric acid)**

|  | **AUC** | **95%CI low** | **95%CI upp** | **Cut off Value** | **Specificity** | **Sensitivity** |
| --- | --- | --- | --- | --- | --- | --- |
| **HTN alone** |  |  |  |  |  |  |
| AIP | 0.52 | 0.51 | 0.53 | -0.30 | 0.23 | 0.81 |
| LAP | 0.59 | 0.59 | 0.60 | 31.66 | 0.42 | 0.73 |
| VAI | 0.55 | 0.54 | 0.56 | 1.02 | 0.34 | 0.74 |
| TyG | 0.58 | 0.57 | 0.59 | 8.62 | 0.56 | 0.56 |
| BRI | 0.63 | 0.62 | 0.64 | 4.62 | 0.49 | 0.71 |
| ABSI | 0.63 | 0.62 | 0.63 | 0.08 | 0.56 | 0.62 |
| CMI | 0.54 | 0.54 | 0.55 | 0.67 | 0.27 | 0.81 |
| **HUA alone** |  |  |  |  |  |  |
| AIP | 0.63 | 0.62 | 0.64 | -0.01 | 0.57 | 0.62 |
| LAP | 0.58 | 0.57 | 0.59 | 39.36 | 0.49 | 0.64 |
| VAI | 0.57 | 0.56 | 0.58 | 1.35 | 0.47 | 0.63 |
| TyG | 0.58 | 0.57 | 0.59 | 8.57 | 0.51 | 0.62 |
| BRI | 0.52 | 0.50 | 0.53 | 3.02 | 0.15 | 0.90 |
| ABSI | 0.52 | 0.51 | 0.53 | 0.08 | 0.30 | 0.77 |
| CMI | 0.62 | 0.61 | 0.63 | 1.10 | 0.49 | 0.69 |
| **HTN-HUA** |  |  |  |  |  |  |
| AIP | 0.65 | 0.64 | 0.66 | 0.05 | 0.65 | 0.57 |
| LAP | 0.71 | 0.70 | 0.72 | 45.90 | 0.59 | 0.73 |
| VAI | 0.64 | 0.63 | 0.65 | 1.61 | 0.58 | 0.63 |
| TyG | 0.67 | 0.66 | 0.68 | 8.63 | 0.57 | 0.68 |
| BRI | 0.70 | 0.69 | 0.71 | 4.88 | 0.53 | 0.77 |
| ABSI | 0.66 | 0.65 | 0.67 | 0.08 | 0.52 | 0.71 |
| CMI | 0.68 | 0.67 | 0.69 | 1.40 | 0.61 | 0.65 |

**Abbreviations:** AUC, area under the curve; CI, confidence interval; HTN, hypertension; HUA, hyperuricemia; HTN-HUA, hypertension plus hyperuricemia; AIP, atherogenic index of plasma; LAP, lipid accumulation product; VAI, visceral adiposity index; TyG, triglyceride-glucose index; BRI, body roundness index; ABSI, a body shape index; CMI, cardiometabolic index.

**Table S4. Area under the curve and cut off values of seven anthropometric indexes for prediction of HTN alone in different sexes**

|  | **AUC** | **95%CI low** | **95%CI upp** | **Cut off Value** | **Specificity** | **Sensitivity** |
| --- | --- | --- | --- | --- | --- | --- |
| **Male** |  |  |  |  |  |  |
| AIP | 0.51 | 0.50 | 0.52 | -0.06 | 0.42 | 0.60 |
| LAP | 0.58 | 0.56 | 0.59 | 22.20 | 0.28 | 0.84 |
| VAI | 0.52 | 0.51 | 0.53 | 1.01 | 0.35 | 0.69 |
| TyG | 0.56 | 0.55 | 0.57 | 8.62 | 0.50 | 0.59 |
| BRI | 0.62 | 0.61 | 0.64 | 4.53 | 0.51 | 0.68 |
| ABSI | 0.66 | 0.65 | 0.67 | 0.08 | 0.59 | 0.65 |
| CMI | 0.53 | 0.52 | 0.54 | 1.10 | 0.43 | 0.63 |
| **Female** |  |  |  |  |  |  |
| AIP | 0.55 | 0.54 | 0.56 | -0.25 | 0.36 | 0.72 |
| LAP | 0.61 | 0.60 | 0.62 | 31.60 | 0.44 | 0.75 |
| VAI | 0.56 | 0.55 | 0.57 | 1.29 | 0.45 | 0.65 |
| TyG | 0.61 | 0.60 | 0.62 | 8.40 | 0.49 | 0.68 |
| BRI | 0.61 | 0.60 | 0.62 | 4.57 | 0.44 | 0.74 |
| ABSI | 0.61 | 0.60 | 0.62 | 0.08 | 0.68 | 0.48 |
| CMI | 0.57 | 0.56 | 0.58 | 0.65 | 0.31 | 0.80 |

**Abbreviations:** AUC, area under the curve; CI, confidence interval; HTN, hypertension; HUA, hyperuricemia; HTN-HUA, hypertension plus hyperuricemia; AIP, atherogenic index of plasma; LAP, lipid accumulation product; VAI, visceral adiposity index; TyG, triglyceride-glucose index; BRI, body roundness index; ABSI, a body shape index; CMI, cardiometabolic index.

**Table S5. Area under the curve and cut off values of seven anthropometric indexes for prediction of HUA alone in different sexes**

|  | **AUC** | **95%CI low** | **95%CI upp** | **Cut off Value** | **Specificity** | **Sensitivity** |
| --- | --- | --- | --- | --- | --- | --- |
| **Male** |  |  |  |  |  |  |
| AIP | 0.59 | 0.58 | 0.61 | -0.01 | 0.49 | 0.66 |
| LAP | 0.60 | 0.58 | 0.61 | 42.75 | 0.52 | 0.63 |
| VAI | 0.59 | 0.57 | 0.60 | 1.36 | 0.50 | 0.64 |
| TyG | 0.56 | 0.54 | 0.57 | 8.58 | 0.47 | 0.64 |
| BRI | 0.56 | 0.55 | 0.58 | 4.49 | 0.46 | 0.64 |
| ABSI | 0.56 | 0.55 | 0.58 | 0.08 | 0.41 | 0.71 |
| CMI | 0.60 | 0.58 | 0.61 | 1.11 | 0.43 | 0.72 |
| **Female** |  |  |  |  |  |  |
| AIP | 0.63 | 0.61 | 0.65 | -0.03 | 0.62 | 0.60 |
| LAP | 0.66 | 0.64 | 0.68 | 45.09 | 0.56 | 0.70 |
| VAI | 0.63 | 0.61 | 0.65 | 1.78 | 0.60 | 0.62 |
| TyG | 0.60 | 0.58 | 0.62 | 8.60 | 0.57 | 0.60 |
| BRI | 0.65 | 0.63 | 0.67 | 5.44 | 0.55 | 0.68 |
| ABSI | 0.52 | 0.50 | 0.54 | 0.08 | 0.51 | 0.54 |
| CMI | 0.65 | 0.63 | 0.67 | 1.00 | 0.50 | 0.75 |

**Abbreviations:** AUC, area under the curve; CI, confidence interval; HTN, hypertension; HUA, hyperuricemia; HTN-HUA, hypertension plus hyperuricemia; AIP, atherogenic index of plasma; LAP, lipid accumulation product; VAI, visceral adiposity index; TyG, triglyceride-glucose index; BRI, body roundness index; ABSI, a body shape index; CMI, cardiometabolic index.

**Table S6. Area under the curve and cut off values of seven anthropometric indexes for prediction of HTN-HUA in different sexes**

|  | **AUC** | **95%CI low** | **95%CI upp** | **Cut off Value** | **Specificity** | **Sensitivity** |
| --- | --- | --- | --- | --- | --- | --- |
| **Male** |  |  |  |  |  |  |
| AIP | 0.63 | 0.61 | 0.64 | 0.05 | 0.57 | 0.62 |
| LAP | 0.70 | 0.69 | 0.72 | 43.32 | 0.55 | 0.76 |
| VAI | 0.64 | 0.62 | 0.65 | 1.63 | 0.60 | 0.61 |
| TyG | 0.64 | 0.63 | 0.66 | 8.75 | 0.58 | 0.62 |
| BRI | 0.72 | 0.71 | 0.74 | 4.88 | 0.58 | 0.75 |
| ABSI | 0.65 | 0.64 | 0.67 | 0.08 | 0.48 | 0.74 |
| CMI | 0.66 | 0.64 | 0.67 | 1.53 | 0.58 | 0.65 |
| **Female** |  |  |  |  |  |  |
| AIP | 0.66 | 0.65 | 0.68 | -0.10 | 0.55 | 0.70 |
| LAP | 0.74 | 0.72 | 0.75 | 46.21 | 0.60 | 0.75 |
| VAI | 0.67 | 0.66 | 0.68 | 1.49 | 0.53 | 0.73 |
| TyG | 0.70 | 0.69 | 0.72 | 8.61 | 0.61 | 0.70 |
| BRI | 0.73 | 0.72 | 0.75 | 5.71 | 0.62 | 0.73 |
| ABSI | 0.63 | 0.61 | 0.65 | 0.08 | 0.59 | 0.62 |
| CMI | 0.70 | 0.68 | 0.71 | 1.05 | 0.54 | 0.75 |

**Abbreviations:** AUC, area under the curve; CI, confidence interval; HTN, hypertension; HUA, hyperuricemia; HTN-HUA, hypertension plus hyperuricemia; AIP, atherogenic index of plasma; LAP, lipid accumulation product; VAI, visceral adiposity index; TyG, triglyceride-glucose index; BRI, body roundness index; ABSI, a body shape index; CMI, cardiometabolic index.

**Table S7. Mean area under the curve and cut-off values of seven anthropometric indexes for prediction of HTN alone, HUA alone and HTN-HUA (after 500 times bootstrap resampling)**

|  | **AUC** | **95%CI low** | **95%CI upp** | **Cut off Value** | **Specificity** | **Sensitivity** |
| --- | --- | --- | --- | --- | --- | --- |
| **HTN alone** |  |  |  |  |  |  |
| AIP | 0.53 | 0.52 | 0.54 | -0.17 | 0.37 | 0.68 |
| LAP | 0.59 | 0.58 | 0.60 | 31.60 | 0.42 | 0.73 |
| VAI | 0.54 | 0.53 | 0.55 | 1.02 | 0.34 | 0.73 |
| TyG | 0.58 | 0.57 | 0.59 | 8.57 | 0.53 | 0.59 |
| BRI | 0.61 | 0.61 | 0.62 | 4.53 | 0.47 | 0.72 |
| ABSI | 0.63 | 0.62 | 0.64 | 0.08 | 0.65 | 0.55 |
| CMI | 0.55 | 0.54 | 0.56 | 0.92 | 0.41 | 0.67 |
| **HUA alone** |  |  |  |  |  |  |
| AIP | 0.61 | 0.60 | 0.63 | -0.01 | 0.56 | 0.63 |
| LAP | 0.61 | 0.60 | 0.63 | 43.90 | 0.54 | 0.65 |
| VAI | 0.59 | 0.58 | 0.61 | 1.46 | 0.52 | 0.64 |
| TyG | 0.57 | 0.56 | 0.59 | 8.58 | 0.52 | 0.63 |
| BRI | 0.58 | 0.57 | 0.59 | 4.72 | 0.47 | 0.65 |
| ABSI | 0.52 | 0.51 | 0.53 | 0.08 | 0.26 | 0.79 |
| CMI | 0.62 | 0.61 | 0.63 | 1.10 | 0.49 | 0.71 |
| **HTN-HUA** |  |  |  |  |  |  |
| AIP | 0.63 | 0.62 | 0.64 | -0.01 | 0.58 | 0.63 |
| LAP | 0.72 | 0.71 | 0.73 | 43.32 | 0.56 | 0.77 |
| VAI | 0.65 | 0.64 | 0.66 | 1.61 | 0.58 | 0.65 |
| TyG | 0.66 | 0.65 | 0.67 | 8.63 | 0.57 | 0.68 |
| BRI | 0.73 | 0.72 | 0.74 | 5.23 | 0.60 | 0.73 |
| ABSI | 0.64 | 0.62 | 0.65 | 0.08 | 0.54 | 0.67 |
| CMI | 0.67 | 0.66 | 0.68 | 1.17 | 0.53 | 0.73 |

**Abbreviations:** AUC, area under the curve; CI, confidence interval; HTN, hypertension; HUA, hyperuricemia; HTN-HUA, hypertension plus hyperuricemia; AIP, atherogenic index of plasma; LAP, lipid accumulation product; VAI, visceral adiposity index; TyG, triglyceride-glucose index; BRI, body roundness index; ABSI, a body shape index; CMI, cardiometabolic index.


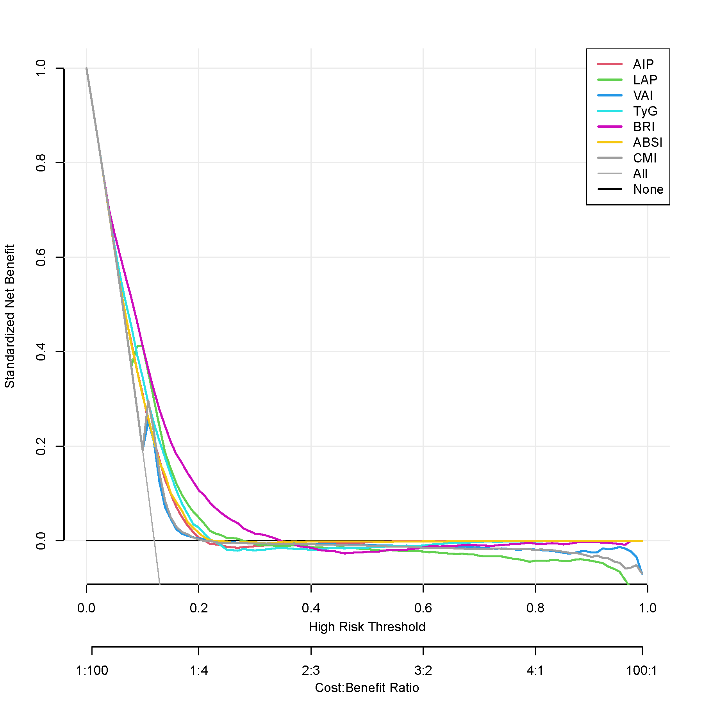


**Figure S1.** DCA for seven anthropometric indexes to predict HTN-HUA.

**Abbreviations:** DCA, decision curve analysis; HTN, hypertension; HUA, hyperuricemia; HTN-HUA, hypertension plus hyperuricemia; ABSI, a body shape index; BRI, body roundness index; LAP, lipid accumulation product; TyG, triglyceride-glucose index; CMI, cardiometabolic index; VAI, visceral adiposity index; AIP, atherogenic index of plasma.


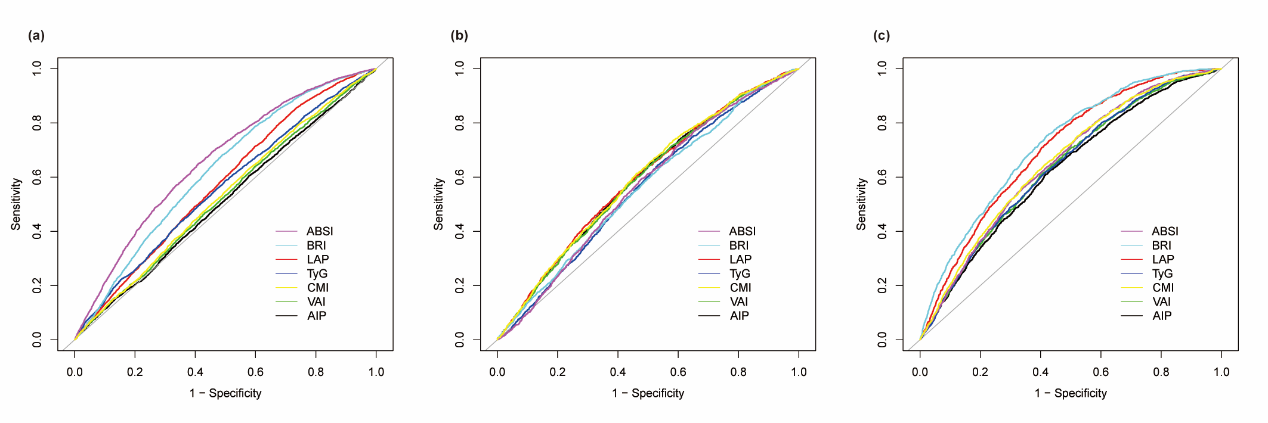


**Figure S2.** Receiver operating characteristic curves for seven anthropometric indexes to predict (a) HTN alone; (b) HUA alone; and (c) HTN-HUA in males.

**Abbreviations:** HTN, hypertension; HUA, hyperuricemia; HTN-HUA, hypertension plus hyperuricemia; ABSI, a body shape index; BRI, body roundness index; LAP, lipid accumulation product; TyG, triglyceride-glucose index; CMI, cardiometabolic index; VAI, visceral adiposity index; AIP, atherogenic index of plasma.


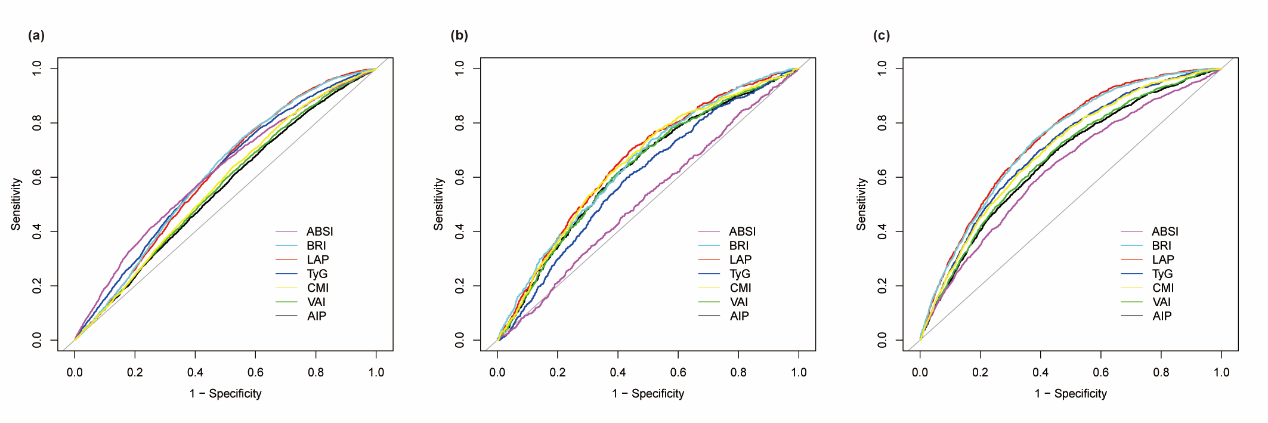


**Figure S3.** Receiver operating characteristic curves for seven anthropometric indexes to predict (a) HTN alone; (b) HUA alone; and (c) HTN-HUA in females.

**Abbreviations:** HTN, hypertension; HUA, hyperuricemia; HTN-HUA, hypertension plus hyperuricemia; ABSI, a body shape index; BRI, body roundness index; LAP, lipid accumulation product; TyG, triglyceride-glucose index; CMI, cardiometabolic index; VAI, visceral adiposity index; AIP, atherogenic index of plasma.


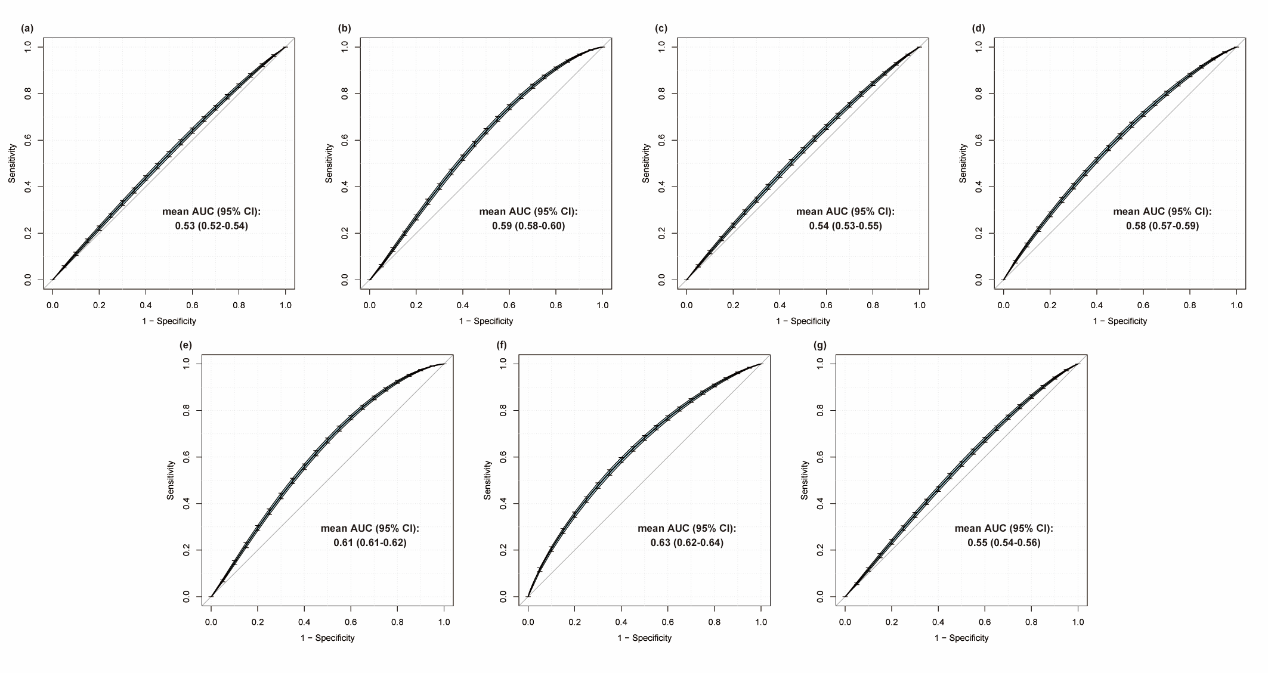


**Figure S4.** Receiver operating characteristic curves by using bootstrap resampling (times = 500) for seven anthropometric indexes ((a)AIP; (b)LAP; (c)VAI; (d)TyG; (e)BRI; (f)ABSI; (g)CMI) to predict HTN alone. Shading shows the bootstrap estimated 95% confidence intervals with the area under the curve.

**Abbreviations:** HTN, hypertension; AIP, atherogenic index of plasma; LAP, lipid accumulation product; VAI, visceral adiposity index; TyG, triglyceride-glucose index; BRI, body roundness index; ABSI, a body shape index; CMI, cardiometabolic index.


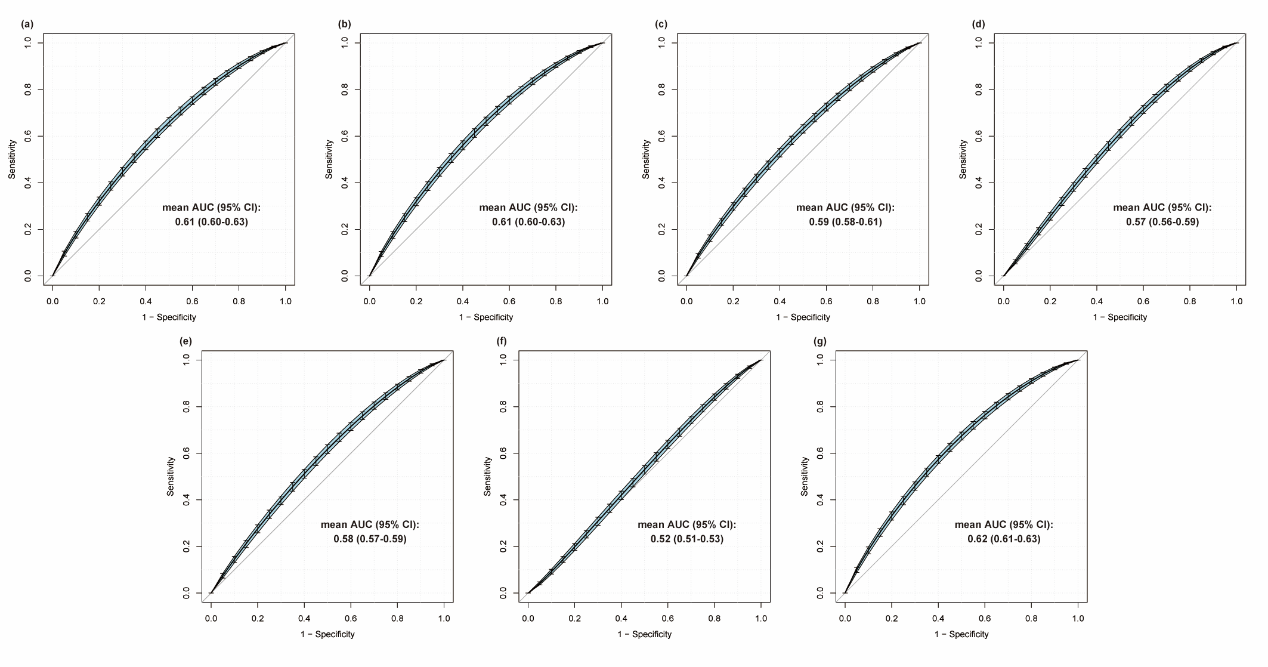


**Figure S5.** Receiver operating characteristic curves by using bootstrap resampling (times = 500) for seven anthropometric indexes ((a)AIP; (b)LAP; (c)VAI; (d)TyG; (e)BRI; (f)ABSI; (g)CMI) to predict HUA alone. Shading shows the bootstrap estimated 95% confidence intervals with the area under the curve.

**Abbreviations:** HUA, hyperuricemia; AIP, atherogenic index of plasma; LAP, lipid accumulation product; VAI, visceral adiposity index; TyG, triglyceride-glucose index; BRI, body roundness index; ABSI, a body shape index; CMI, cardiometabolic index.


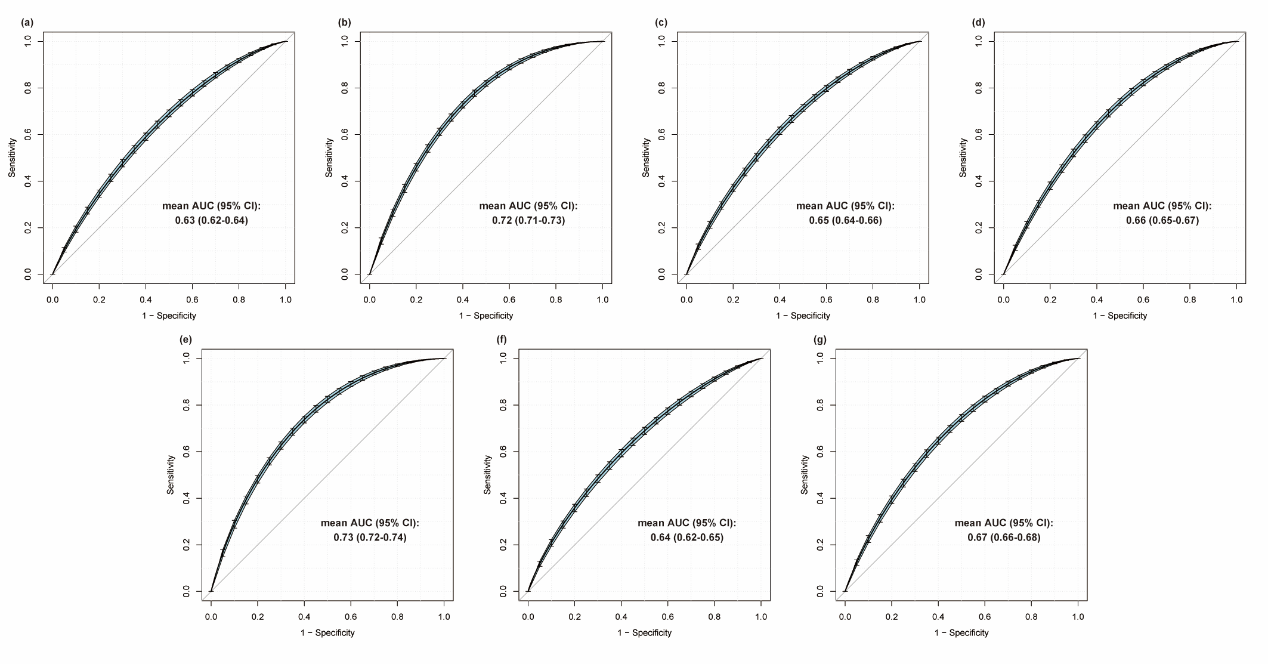


**Figure S6.** Receiver operating characteristic curves by using bootstrap resampling (times = 500) for seven anthropometric indexes ((a)AIP; (b)LAP; (c)VAI; (d)TyG; (e)BRI; (f)ABSI; (g)CMI) to predict HTN-HUA. Shading shows the bootstrap estimated 95% confidence intervals with the area under the curve.

**Abbreviations:** HTN-HUA, hypertension plus hyperuricemia; AIP, atherogenic index of plasma; LAP, lipid accumulation product; VAI, visceral adiposity index; TyG, triglyceride-glucose index; BRI, body roundness index; ABSI, a body shape index; CMI, cardiometabolic index.
